# Gene-environment interaction for polymorphisms in ataxia telangiectasia-mutated gene and radiation exposure in carcinogenesis: results from two literature-based meta-analyses of 27120 participants

**DOI:** 10.18632/oncotarget.12724

**Published:** 2016-10-18

**Authors:** Yuguang Zhao, Lei Yang, Di Wu, Hua He, Mengmeng Wang, Tingwen Ge, Yudi Liu, Huimin Tian, Jiuwei Cui, Lin Jia, Ziqiang Wan, Fujun Han

**Affiliations:** ^1^ Cancer Center, The First Hospital of Jilin University, Changchun, China

**Keywords:** ataxia telangiectasia-mutated, carcinogenesis, gene-environment interaction, polymorphism, radiation

## Abstract

**Purpose:**

We conducted two meta-analyses of *ATM* genetic polymorphisms and cancer risk in individuals with or without radiation exposure to determine whether there was a joint effect between the *ATM* gene and radiation exposure in carcinogenesis.

**Results:**

rs1801516, which was the only *ATM* polymorphism investigated by more than 3 studies of radiation exposure, was eligible for the present study. The meta-analysis of 23333 individuals without radiation exposure from 24 studies showed no association between the rs1801516 polymorphism and cancer risk, without heterogeneity across studies. The meta-analysis of 3787 individuals with radiation exposure from 6 studies showed a significant association between the rs1801516 polymorphism and a decreased cancer risk, with heterogeneity across studies. There was a borderline-significant difference between the ORs of the two meta-analyses (*P* = 0.066), and the difference was significant when only Caucasians were included (*P* = 0.011).

**Materials and methods:**

Publications were identified by searching PubMed, EMBASE, Web of Science, and CNKI databases. Odds ratios (ORs) were calculated to estimate the association between *ATM* genetic polymorphisms and cancer risk. Tests of interaction were used to compare differences between the ORs of the two meta-analyses.

**Conclusions:**

Our meta-analyses confirmed the presence of a gene-environment interaction between the rs1801516 polymorphism and radiation exposure in carcinogenesis, whereas no association was found between the rs1801516 polymorphism and cancer risk for individuals without radiation exposure. The heterogeneity observed in the meta-analysis of individuals with radiation exposure might be due to gene-ethnicity or gene-gene interactions. Further studies are needed to elucidate sources of the heterogeneity.

## INTRODUCTION

The International Agency for Research on Cancer has confirmed that ionizing radiation is associated with an increased risk for a wide range of cancers, including breast cancer, thyroid cancer, and leukemia [1]. The risk for carcinogenesis associated with radiation exposure is influenced by genetic background [2, 3]. Understanding gene–environment interactions in carcinogenesis has been a stated priority for the National Cancer Institute [4].

The ataxia telangiectasia-mutated (*ATM*) protein plays a central role in mediating the cellular response to radiation-induced DNA damage [5]. Germ-line inactivating mutations in the *ATM* gene cause ataxia-telangiectasia, a recessive genetic disorder with a high incidence of cancer [6]. Ataxia-telangiectasia heterozygotes appear to have a greater risk of developing cancer than the wild-type homozygotes, leading to the estimation that polymorphisms in the *ATM* gene may alter the risk of carcinogenesis [7]. In the past two decades, about 100 studies have been published to evaluate the associations of *ATM* genetic polymorphisms with cancer risk. Some of the polymorphisms have been reported by more than 10 studies, such as rs1801516, IVS10-6T > G, rs1800057, rs1800054, rs1800056, rs1800058, and rs4986761. Although most of the findings on these polymorphisms were inconsistent, a meta-analysis of 11120 participants showed a significant association between the rs1800057 polymorphism and breast cancer risk [8]. Recently, two meta-analyses demonstrated evidence for gene-environment interactions between the *ATM* gene and radiation exposure in the development of radiotherapy-induced adverse events [9, 10]. Taken together, these suggest a possible role of *ATM* genetic polymorphisms in carcinogenesis through gene–radiation interactions.

A number of studies have investigated the joint effect between the *ATM* gene and radiation exposure on cancer risk. The first study published in 2002 showed that polymorphisms in the *ATM* gene were not associated with an increased breast cancer risk in patients with Hodgkin's disease after radiotherapy [11]. Subsequently, 5 studies have been conducted on this issue, with inconsistent results [12–16]. Given the uncertainty and the lack of a meta-analysis on this topic, we conducted two meta-analyses of *ATM* genetic polymorphisms and cancer risk in individuals in the presence or absence of radiation exposure to determine whether there was a joint effect between the *ATM* gene and radiation exposure in carcinogenesis.

## RESULTS

### Assessing quality of included studies

**Figure 1:**
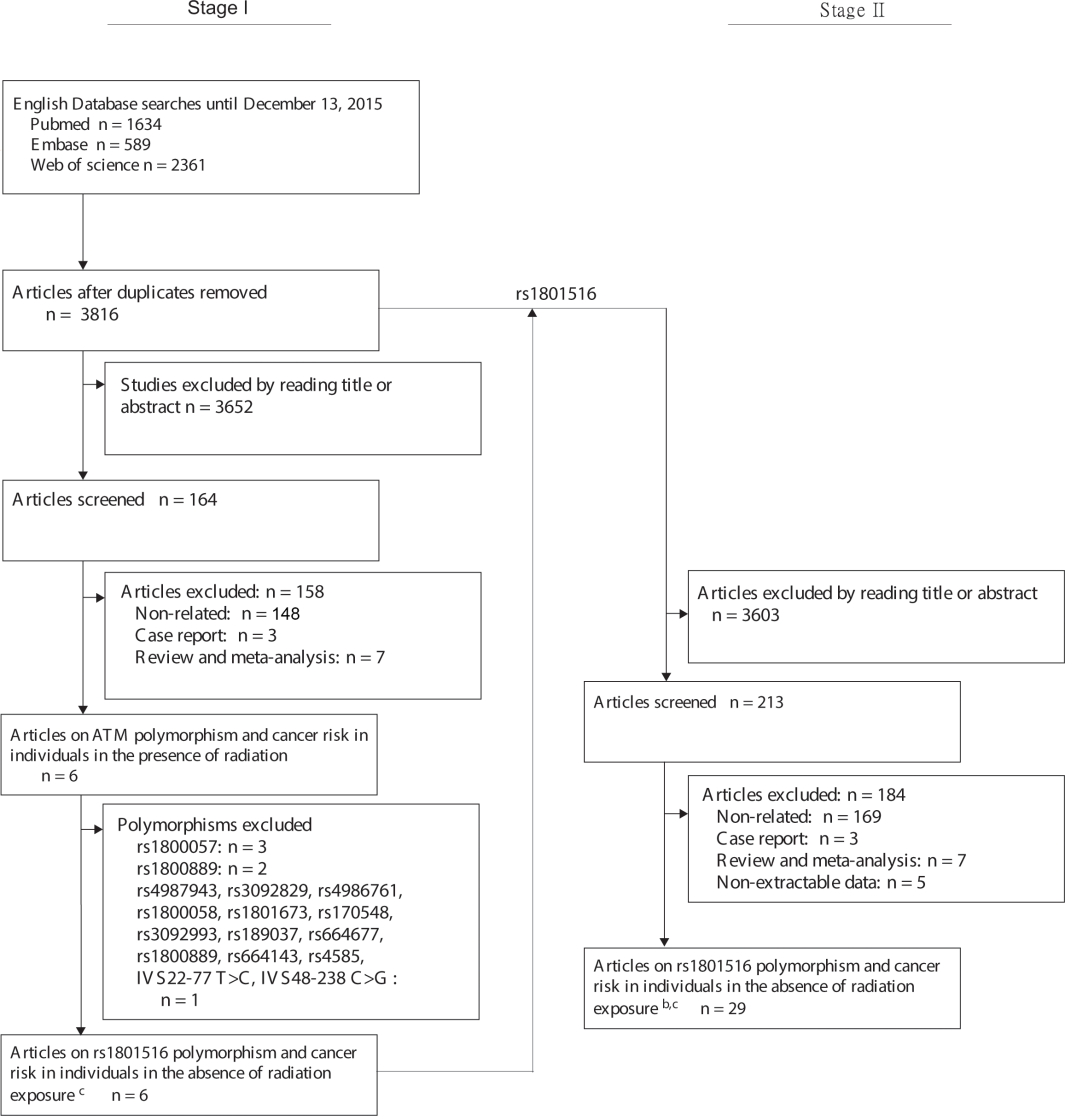
Flow chart for inclusion and exclusion of studies ^a^The search on Chinese National Knowledge Infrastructure (CNKI) database identified no study of the *ATM* rs1801516 polymorphism and cancer risk. ^b^5 studies were identified with methodological errors and were excluded from the present meta-analysis in the subsequent quality assessment procedure [12, 18–21]. ^c^One article reported data for radiation exposed as well as unexposed populations, the results for each group were considered as a separate study [12]

rs1801516 was the only *ATM* genetic polymorphism investigated by more than 3 studies of radiation exposure, and was eligible for the present study. A total of 29 studies were identified for the meta-analysis of individuals without radiation exposure [12, 17–44], and 6 studies for the meta-analysis of individuals with radiation exposure [11–16] (Figure 1). The *ATM* rs1801516 genotype distribution in controls was not in Hardy–Weinberg equilibrium (HWE) in 5 studies [12, 18–21], could not be assessed in 4 studies [11, 13, 25, 26], and was in HWE for the other studies [17, 22–24, 27–44]. As a result, 5 studies were identified with methodological errors and were excluded from a meta-analysis [12, 18–21]. The quality assessments according to Newcastle–Ottawa scale (NOS) [45] were described in Supplementary Table S1. The included studies had a relatively high quality with a median score of 7, ranging from 5 to 9. The quality was high for 22 studies (≥ 6) [11–16, 25, 26, 28–30, 32–40, 42, 43] and low for 8 studies (≤ 5) [17, 22–24, 27, 31, 41, 44].

### Meta-analysis for individuals in the absence of radiation exposure

**Figure 2:**
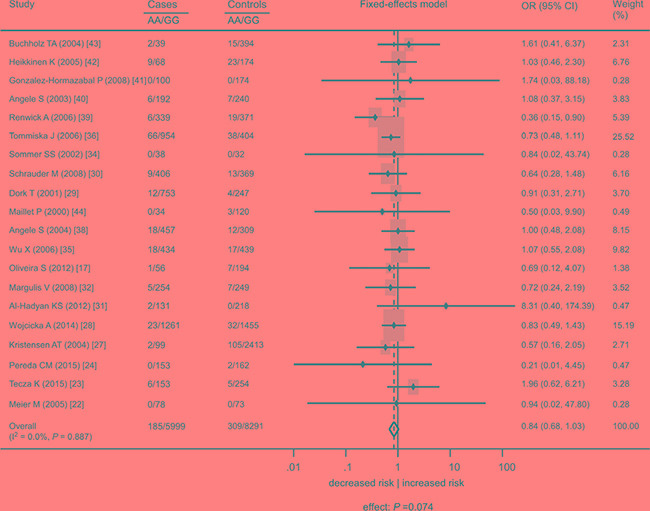
Association between the *ATM* rs1801516 polymorphism and cancer risk in individuals in the absence of radiation exposure under the homozygous model AA represents the number of individuals who carry the AA alleles. GG represents the number of individuals who carry the GG alleles. ORs for each study are represented by the squares, and the horizontal line crossing the square represents the 95% CI. The diamond represents the estimated overall effect based on the meta-analysis. ORs and 95%CIs were computed by applying a continuity correction (addition of 0.5 in all the cells) in order to overcome problems resulted from cells containing zero values [69]. All statistical tests were two sided. Abbreviations: CI, confidence interval; OR, odds ratio

**Figure 3:**
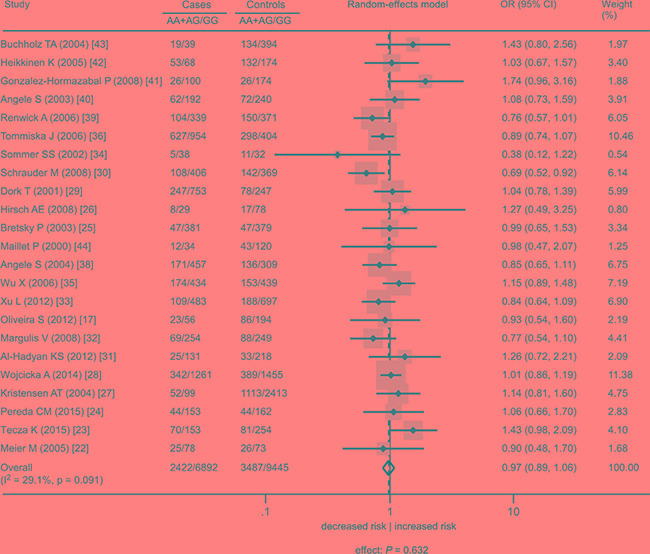
Association between the *ATM* rs1801516 polymorphism and cancer risk in individuals in the absence of radiation exposure under the dominant model AA + AG represents the number of individuals who carry the AA or AG alleles. GG represents the number of individuals who carry the GG alleles. ORs for each study are represented by the squares, and the horizontal line crossing the square represents the 95% CI. The diamond represents the estimated overall effect based on the meta-analysis. All statistical tests were two sided. Abbreviations: CI, confidence interval; OR, odds ratio

This meta-analysis included 24 studies with 9858 cases and 13475 controls [17, 22–44] (Table 1). When all cancer types were considered, there was no significant association of the rs1801516 polymorphism with cancer risk (homozygous model: odds ratio [OR] = 0.84, 95% confidence interval [CI]: 0.68, 1.03, *P* = 0.074; heterozygous model: OR = 0.99, 95%CI: 0.91, 1.07, *P* = 0.784; recessive model: OR = 0.87, 95%CI: 0.69, 1.10, *P* = 0.231; dominant model: OR = 0.97, 95%CI: 0.89, 1.06, *P* = 0.632). There was little evidence of heterogeneity across studies (I^2^ ≤ 29.1%). Subgroup analyses were conducted in order to check whether the features of the included studies affected the results of this meta-analysis. For each genetic model, there was little variation in the effect sizes according to cancer site, ethnicity, study quality, and study size. Figures 2–3 showed the forest plot of the association under the homozygous and dominant models, and Tables 2–3 showed the subgroup analyses under the homozygous and dominant models. The results under the heterozygous and recessive models were similar to those under the dominant and homozygous models, and thus were not shown in figures and tables. For all the meta-analyses, sensitivity analyses did not identify any single study that markedly influenced the estimates, indicating that these results were reliable.

**Table 1:** Characteristics of studies included in the meta-analysis of individuals in the absence of radiation exposure

First author, year [Ref.]	Ethnicity	Region/Country	Type of cancer	Family history of cancer	HWE in controls	Minor allele frequency	Cases/controls
Maillet P, 2000, [44]	Swiss	Switzerland	Colorectal cancer	Yes	Yes	0.14	46/163
Buchholz TA, 2004, [43]	Mixed population (75% Caucasian)	USA	Breast cancer	No	Yes	0.14	58/528
Heikkinen K, 2005, [42]	Finnish	Finland	Breast cancer	Yes	Yes	0.25	121/306
Gonzalez-Hormazabal P, 2008, [41]	Chilean	Chile	Breast cancer	Yes	Yes	0.07	126/200
Angele S, 2003, [40]	NR	France	Breast cancer	No	Yes	0.13	254/312
Renwick A, 2006, [39]	UK ethnic(whites)	UK	Breast cancer	Yes	Yes	0.16	443/521
Angele S, 2004, [38]	Caucasian	UK	Prostate cancer	No	Yes	0.17	628/445
Yang H, 2007, [37]	Caucasian	USA	Non-small cell lung cancer	No	Yes	> 0.05	544/546
Tommiska J, 2006, [36]	Finnish	Finland	Breast cancer	Both	Yes	0.24	1581/702
Wu X, 2006, [35]	Whites (89.3%)	USA	Bladder cancer	No	Yes	0.14	608/592
Sommer SS, 2002, [34]	Caucasian (> 80%)	USA	Breast cancer	No	Yes	0.13	43/43
Xu L, 2012, [33]	Non-hispanic whites; mixed population	USA	Thyroid carcinoma	No	Yes	> 0.10	592/885
Margulis V, 2008, [32]	NR	USA	Renal cancer	No	Yes	0.14	323/337
Al-Hadyan KS, 2012, [31]	NR	Saudi Arabia	Head and neck cancer	No	Yes	0.07	156/251
Schrauder M, 2008, [30]	NR	German	Breast cancer	No	Yes	0.15	514/511
Dork T, 2001, [29]	Caucasian	Germany	Breast cancer	No	Yes	0.13	1000/325
Wojcicka A, 2014, [28]	Caucasian	Poland	Thyroid cancer	No	Yes	0.11	1603/1844
Kristensen AT, 2004, [27]	NR	Norway	Rectal cancer	No	Yes	0.17	151/3526
Hirsch AE, 2008, [26]	African-American	USA	Breast cancer	No	NR	> 0.05	37/95
Bretsky P, 2003, [25]	African-American, Latina,Japanese, and Caucasian	USA	Breast cancer	No	NR	> 0.03	428/426
Pereda CM, 2015, [24]	mixed	Cuban	Thyroid cancer	No	Yes	0.11	197/206
Tecza K, 2015, [23]	Caucasian	Poland	Ovarian cancer	No	Yes	0.13	223/335
Meier M, 2005, [22]	Caucasian	Germany	T cell acute lymphoblastic leukemia	No	Yes	0.13	103/96
Oliveira S, 2012, [17]	Portuguese	Portugal	Cervical cancer	No	Yes	0.17	79/280

Abbreviations: HWE, Hardy–Weinberg equilibrium.

**Table 2:** Subgroup analyses for the association between the ATM rs1801516 polymorphism and cancer risk in individuals in the absence of radiation exposure under the homozygous model

Study selection	Studies (*n*)	Cases	Controls	Heterogeneity		Effect	
		AA/GG	AA/GG	I^2^ (%)	*P* value	OR (95%CI)	*P* value
Quality score							
≥ 6	12	174/5195	187/4683	0.0	0.858	0.81 (0.65–1.01)	0.060
≤ 5	8	11/804	122/3608	0.0	0.617	0.99 (0.53–1.83)	0.976
Sample size							
Large (> 500)	12	173/5341	274/7144	0.0	0.675	0.82 (0.66–1.02)	0.081
Small (< 500)	8	12/658	35/1147	0.0	0.909	0.98 (0.52–1.86)	0.962
Family history of cases^b^							
Sporadic cancer	16	137/4989	264/7452	0.0	0.960	0.88 (0.70–1.11)	0.269
Family cancer	5	48/1010	83/1243	4.1	0.947	0.71 (0.49–1.03)	0.071
Ethnicity							
Caucasion	17	183/5615	307/7737	0.0	0.941	0.82 (0.67–1.02)	0.066
Site							
Breast	9	110/2889	119/2404	0.0	0.704	0.76 (0.57–1.01)	0.060
Sum	20	185/5999	309/8291	0.0	0.887	0.84 (0.68–1.03)	0.074

AA represents the number of individuals who carry the AA alleles. GG represents the number of individuals who carry the GG alleles. Abbreviations: CI, confidence interval; HWE, Hardy–Weinberg equilibrium; OR, odds ratio.

a The genotype distribution in controls was in HWE in all the studies.

b The study by Tommiska et al. [36] reported the risks of both familial and sporadic cancer in comparison with the same controls, and the results for each were considered as a separate study.

**Table 3:** Subgroup analyses for the association between the *ATM* rs1801516 polymorphism and cancer risk in individuals in the absence of radiation exposure under the dominant model

Study selection	Studies (*n*)	Cases	Controls	Heterogeneity		Effect	
		AA + AG/GG	AA + AG/GG	I^2^ (%)	*P* value	OR (95% CI)	*P* value
Quality score							
≥ 6	15	2145/6088	2035/5837	32.4	0.103	0.92 (0.85–1.00)	0.054
≤ 5	8	277/804	1452/3608	0.0	0.705	1.18 (1.00–1.41)	0.058
Sample size							
Large (> 500)	14	2201/6205	3069/8220	0.052	41.5	0.95 (0.86–1.05)	0.325
Small (< 500)	9	221/687	418/1225	0.573	0.0	1.06 (0.87–1.30)	0.536
Family history of cases^a^							
Familial cancer	5	504/1010	649/1243	0.169	37.8	0.91 (0.79–1.06)	0.214
Sporadic cancer	19	1902/5665	3115/8428	0.170	23.6	0.97 (0.90–1.04)	0.352
Ethnicity							
Caucasian	19	2279/6068	3320/8345	0.126	27.9	0.95 (0.88–1.02)	0.114
Site							
Breast	11	1306/3299	1107/2862	0.085	39.5	0.95 (0.81–1.10)	0.462
Thyroid	3	495/1897	621/2314	0.304	16.1	0.96 (0.84–1.10)	0.571
HWE in controls							
Yes	21	2367/6482	3423/8988	0.073	32.9	0.96 (0.89–1.03)	0.640
Overall	23	2422/6892	3487/9445	0.114	27.1	0.97 (0.89–1.06)	0.632

AA + AG represents the number of individuals who carry the AA or AG alleles. GG represents the number of individuals who carry the GG alleles. Abbreviations: CI, confidence interval; HWE, Hardy–Weinberg equilibrium; OR, odds ratio.

a The study by Tommiska et al. [36] reported the risks of both familial and sporadic cancer in comparison with the same controls, and the results for each were considered as a separate study.

We examined if there was evidence of publication bias for each meta-analysis that included 10 or more studies. Asymmetry in the funnel plots was not observed under any comparisons, and significant asymmetry was not suggested by Egger's linear regression test or Begg's rank correlation test (Supplementary Figure S1).

### Meta-analysis for individuals in the presence of radiation exposure

There were 6 studies with 1459 cases and 2328 controls eligible for this meta-analysis [11–16]. The main characteristics of these studies were presented in Table 4. 2 out of 6 studies investigated the association between the rs1801516 polymorphism and contralateral breast cancer risk in breast cancer patients after radiotherapy [13, 14], 1 study investigated the association between the rs1801516 polymorphism and breast cancer risk in patients with Hodgkin's disease after radiotherapy [11], and 3 studies investigated the association between the rs1801516 polymorphism and papillary thyroid carcinoma risk in individuals who lived in the areas contaminated by radionuclides [12, 15, 16]. 5 out of 6 studies were conducted in Caucasians [11–15], and 1 in Polynesians [16]. All the included studies had used histologic analyses to confirm cancers.

**Table 4:** Characteristics of studies included in the meta-analysis of individuals in the presence of radiation exposure

**First author, year [Ref.]**	**Ethnicity**	**Region/Country**	**Investigation arm**	**Control arm**	**Family history of cases**	**HWE in controls**	**Minor allele frequency**	**Cases/controls**
Akulevich NM, 2009, [12]^a^	Caucasian	European part of Russia	IR-induced thyroid cancer (Cases lived in the areas contaminated with radionuclides from Chernobyl fallouts; the cases were younger than 15 years at the time of the Chernobyl accident; The median time to develop PTC was 14 years.)	IR-exposed controls (the controls were matched to the cases by age and geographic region.)	No	Yes	0.17	122/198
Damiola F, 2014, [15]^a^	Caucasian	Belarus	IR-induced thyroid cancer (cases lived in the areas contaminated with radionuclides from Chernobyl fallouts. At the time of the Chernobyl accident, the cases were younger than 18 years old; the cases were diagnosed within 6–12 years after the accident.)	IR-exposed controls (residents of the same settlements as the cases. Age of IR-exposed controls was set to be ± 3 years of the cases.)	No	Yes	0.16	70/250
Broeks A, 2008, [13]	Caucasian	Netherlands	Therapy-induced contralateral breast cancer (the first breast cancer was diagnosed before age 50. There is an interval of at least 1 year between the first and the second breast cancer.)	Unilateral breast cancer (the first breast cancer was diagnosed before age 50. The patients were disease-free of a second breast cancer for at least 5 years.)	No	NR	> 0.10	247/190
Concannon P, 2008, [14]	Caucasian	USA	Therapy-induced contralateral breast cancer (the first breast cancer was diagnosed before age 55. There is an interval of at least 1 year between the first and the second breast cancer. Median interval between first diagnosis and reference date was 4.3 years.)	Unilateral breast cancer (the first breast cancer was diagnosed before age 55. The patients were disease-free of a second breast cancer for at least 1 year. Median interval between first diagnosis and reference date was 4.3 years.)	No	Yes	0.13	808/1397
Offit K, 2002, [11]	Caucasian	USA	Radiation-induced breast cancer after treatment for Hodgkin's disease (The median time to develop breast cancer was 18 years.)	Patients with Hodgkin's disease who did not develop breast cancer (The median follow-up was 17 years.)	No	NR	NR	37/23
Maillard S, 2015, [16]	Polynesian	France	IR-induced thyroid cancer (Cases lived in the areas where a total of 41 atmospheric nuclear weapons tests were carried out between 1966 and 1974 and where individuals were at an increased risk of developing thyroid cancer caused by radionuclides [74]. All cases were under the age of 15 in 1974, and all were diagnosed for thyroid cancer between 1979 and 2004. Age distribution was ranged from 10 to 62.)	IR-exposed controls (the controls were matched to the cases by race, age and geographic region.)	No	Yes	0.02	175/270

Abbreviations: HWE, Hardy–Weinberg equilibrium; IR, ionizing radiation.

a There is no overlap in the participants between the two studies [12, 15].

**Figure 4:**
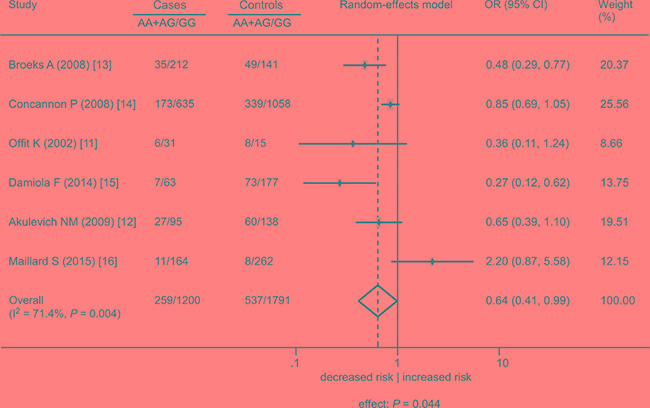
Association between the *ATM* rs1801516 polymorphism and cancer risk in individuals in the presence of radiation exposure under the dominant model AA + AG represents the number of individuals who carry the AA or AG alleles. GG represents the number of individuals who carry the GG alleles. ORs for each study are represented by the squares, and the horizontal line crossing the square represents the 95% CI. The diamond represents the estimated overall effect based on the meta-analysis. All statistical tests were two sided. Abbreviations: CI, confidence interval; OR, odds ratio

To include all 6 studies for a summary OR estimate, the meta-analysis could only be conducted under the dominant model. The result showed a significant association between the rs1801516 polymorphism and a decreased risk of radiation-induced cancer (OR = 0.64, 95% CI: 0.41, 0.99; *P* = 0.044), with high between study heterogeneity (I^2^ = 71.4%, *P* = 0.004) (Figure 4). Sensitivity analyses identified that the study by Maillard et al. was the outlier, and the association was more significant after this study was excluded (OR = 0.55, 95% CI: 0.36, 0.83; *P* = 0.005) [16]. However, the heterogeneity remained significant (I^2^ = 66.9%, *P* = 0.017), indicating that other factors might contribute to the heterogeneity. Table 5 showed the results of the subgroup analyses. A significant association was shown among Caucasians (OR = 0.55, 95% CI: 0.36, 0.83; *P* = 0.005), whereas no association was shown among other subgroups. In addition, there was obvious evidence of heterogeneity in all subgroups (I^2^ ranged 66.9% to 81.8%), suggesting that the examined factors had a minimal influence on the variation of the estimates.

**Table 5:** Subgroup analyses for the association between the *ATM* rs1801516 polymorphism and cancer risk in individuals in the presence of radiation exposure under the dominant model

Study selection	Studies (*n*)	Cases	Controls	Heterogeneity		Effect	
		AA + AG/GG	AA + AG/GG	I^2^ (%)	*P* value	OR (95%CI)	*P* value
Sample size							
Small (< 500)	4	86/565	198/733	68.1	0.014	0.58 (0.33–1.03)	0.065
HWE in controls							
Yes	4	218/857	480/1635	75.2	0.007	0.75 (0.43–1.30)	0.300
Ethnicity							
Caucasion	5	248/1036	529/1529	66.9	0.017	0.55 (0.36–0.83)	0.005
Site							
Breast	3	214/878	396/1214	67.5	0.046	0.61 (0.37–1.03)	0.063
Thyroid	3	45/322	141/577	81.8	0.004	0.71 (0.26–1.97)	0.511
Sum	6	259/1200	537/1791	71.4	0.004	0.64 (0.41–0.99)	0.044

AA + AG represents the number of individuals who carry the AA or AG alleles. GG represents the number of individuals who carry the GG alleles.

Abbreviations: CI, confidence interval; HWE, Hardy–Weinberg equilibrium; OR, odds ratio.

a All the studies included in these analyses were scored as high quality, and all the participants included were classified as sporadic groups.

### Differences in the effect estimates between individuals in the presence or absence of radiation exposure

**Figure 5:**
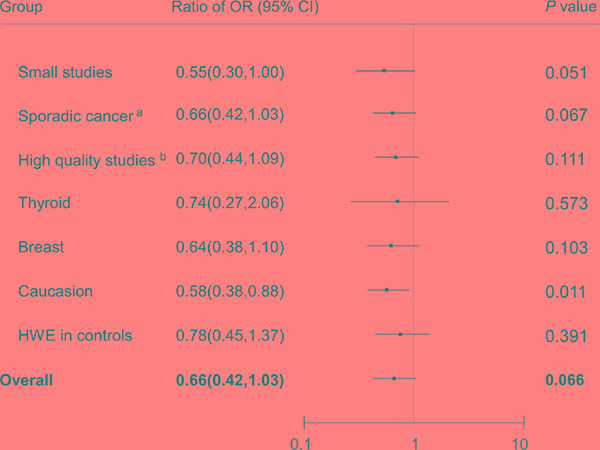
Odds ratios from the meta-analyses of individuals in the presence of radiation exposure were compared with odds ratios from the meta-analyses of individuals in the absence of radiation exposure (dominant model) ORs for each group are represented by the squares, and the horizontal line crossing the square represents the 95% CI. All statistical tests were two sided. Abbreviations: CI, confidence interval; OR, odds ratio. ^a^All the participants included in the meta-analysis of individuals in the presence of radiation exposure were classified as sporadic groups. ^b^All the studies included in the meta-analysis of individuals in the presence of radiation exposure were scored as high quality

The effect estimates for individuals in the absence and presence of radiation exposure were compared to determine the relationship of the interaction (synergistic or antagonistic) between radiation exposure and the rs1801516 polymorphism in carcinogenesis. Figure 5 displayed the comparisons of the ORs between the main meta-analyses and between the subgroup analyses under the dominant model. The genetic effect for all participants in the presence of radiation exposure was borderline significantly larger than that for all participants in the absence of radiation exposure (radio of OR = 0.66, 95% CI: 0.42, 1.03; *P* = 0.066). The difference was statistically significant when only Caucasians were included (radio of OR = 0.58, 95% CI: 0.38, 0.88; *P* = 0.011).

## DISCUSSION

This work represents the first comprehensive assessment of the literature on the gene-environment interaction for polymorphisms in the *ATM* gene and radiation exposure in carcinogenesis. rs1801516, which was the only *ATM* genetic polymorphism investigated by more than 3 studies, was eligible for the present study. Our meta-analyses showed that the rs1801516 polymorphism interacted with radiation exposure, resulting in a synergistic effect in carcinogenesis. In addition, we showed convincing evidence of no association between the rs1801516 polymorphism and cancer risk for individuals in the absence of radiation exposure.

The present meta-analysis of 23333 participants in the absence of radiation exposure had a very large sample size, and was able to provide convincing evidence of no association between the rs1801516 polymorphism and cancer risk. Up to now, 5 meta-analyses have been performed for the role of the rs1801516 polymorphism on cancer risk: 4 on breast cancer [8, 46–48] and 1 on thyroid cancer [49]. One of the meta-analyses showed that homozygous carriers of the rs1801516 genotype had a lower breast cancer risk compared with carriers of the heterozygous and homozygous wild-type genotypes [48]. However, the other studies did not find a significant association between the rs1801516 polymorphism and cancer risk [8, 46, 47, 49]. Compared with the previous meta-analyses [8, 46–49], the present meta-analysis included more studies, and was able to employ rigorous methodology to estimate the genetic effect of the rs1801516 polymorphism on carcinogenesis. The overall meta-analyses of individuals in the absence of radiation exposure showed no association between the rs1801516 polymorphism and cancer risk under the four genetic models. We also conducted subgroup analyses based on cancer site, ethnicity, familial cancer history, study quality, and sample size. For each genetic model, we observed a small variability in the effect sizes between the subgroup analyses and the main meta-analysis. These suggested that the results of the main meta-analysis were independent on the features of the included studies. The extensive consistency provided optimal evidence of the credibility of no association between the rs1801516 polymorphism and cancer risk for individuals in the absence of radiation exposure.

Our meta-analysis of 3787 participants in the presence of radiation exposure provided evidence of an association between the rs1801516 polymorphism and a decreased cancer risk for individuals who exposed to radiation. This meta-analysis included 6 studies across two ethnicities: 1 study in Polynesians and 5 studies in Caucasions. The natures of the two populations are different: the Polynesians are geographically isolated from the rest of the world, and have a significant variation in allele frequencies (minor allele frequency [MAF] in Polynesians = 0.02) as compared to the Caucasians (MAF in Caucasians = 0.19) [16]. The study in Polynesians showed that the minor allele carriers of the rs1801516 polymorphism were associated with an increased cancer risk compared with the main allele carriers in the presence of radiation exposure [16]. On the contrary, all the other studies (Caucasians) showed a consistently decreased cancer risk of the minor allele carriers compared with the main allele carriers in the presence of radiation exposure (2 of 5 comparisons were individually significant [13, 15]). In addition, the test of interaction showed a significant difference in the effect estimates between Caucasions in the presence and absence of radiation exposure. Furthermore, two meta-analyses demonstrated convincing evidence of an association between the rs1801516 polymorphism and radiotherapy-induced adverse events [9, 10]. Taken together, these suggested a gene-environment interaction between the rs1801516 polymorphism and radiation exposure in carcinogenesis, and the interaction might be modified by ethnicity. However, we could not rule out the possibility that the observed association between the rs1801516 polymorphism and cancer risk of Polynesians in the presence of radiation exposure was a chance finding. It should be noted that there was a high variability across studies included in this meta-analysis. Our subgroup analyses failed to explain the heterogeneity, indicating that the study-level factors examined had little influence on the variation of the estimates.

The *ATM* rs1801516 polymorphism is a polymorphic G-to-A transition at nucleotide 5557 of exon 39, resulting in a change from aspartic acid to asparagine at amino acid position 1853 of the protein [50]. *In vitro* data showed that human fibroblasts carrying the minor alleles of the rs1801516 polymorphism increased cellular radiosensitivity compared with those carrying the major alleles [51, 52]. Some variants of the *ATM* gene, including the rs1801516 polymorphism, were reported to be associated with a decreased *ATM* expression and a reduced capacity of DNA damage recognition [42, 53]. Based on these data, it was difficult to figure out how this single polymorphism might be associated with a decreased cancer risk for individuals who were exposed to radiation. Instead, a gene-gene interaction of the *ATM* gene with BRCA1 has been reported [28, 52]. Therefore, it could be expected that the polygenic action of unidentified alleles or genes probably played a non-negligible role on the function of the rs1801516 polymorphism. The differences observed between Polynesians and Caucasians regarding the effect of the rs1801516 polymorphism on cancer risk following radiation exposure as well as the clinical heterogeneity were likely to be due to gene-gene interactions.

Our study has a number of possible limitations. 1) Due to fewer than 10 studies in the meta-analysis of individuals with radiation exposure, the publication bias was not tested by the funnel plot, for this method could not obtain enough power in the case [54]. However, based on the Venice criteria that assess cumulative evidence on genetic associations, an OR of > 0.85 or < 1.15 could be easily susceptible to biases, including phenotyping errors, genotyping errors, population stratification, and selective reporting biases [55–57]. This meta-analysis yielded an OR of 0.55, suggesting that this genetic effect was not so vulnerable to biases. 2) Except for the dominant model, other genetic models, such as recessive, heterozygous, and homozygous models, were not examined because of the limited information in the meta-analysis of individuals in the presence of radiation exposure. Therefore, the gene-environment interaction in other genetic models could not be determined. 3) Due to the lack of individual patient data, we were not able to conduct the present meta-analyses based on individual patient data, in which we can: (a) check each study to apply consistent conditions for inclusion and to standardize analysis techniques, and (b) adjust the analyses for covariates (radiation dose, gender, and age). It is especially so for the study by Broeks et al. that reported the significance of *ATM* variants on secondary breast cancer risk after treatment of primary breast cancer [13]. In this study, 32% patients included in the present meta-analysis did not receive radiotherapy [13]. Because the sensitivity analyses showed no difference in the effect estimates after exclusion of this study, we believed that the incomplete data might reduce the power of the analysis but did not bias it. Moreover, literature based meta-anlayses were considered to be often consistent with those based on individual patient data [58], and should not be viewed as “inferior” [59].

In conclusion, the present study gave a clear picture of gene-environment interaction for the *ATM* rs1801516 genotype and radiation exposure in carcinogenesis: there was convincing evidence of no association between the rs1801516 polymorphism and cancer risk of individuals in the absence of radiation exposure; there was evidence of a gene-environment interaction between the rs1801516 polymorphism and radiation exposure in carcinogenesis, and the heterogeneity observed across studies might be due to gender-ethnicity or gene-gene interactions. Further studies are needed to elucidate sources of the heterogeneity.

## MATERIALS AND METHODS

Our meta-analyses were conducted according to the Preferred Reporting Items for Systematic Reviews and Meta-Analyses guidelines [60].

### Selection criteria

To be eligible for inclusion in our meta-analyses, a study had to meet all the following criteria: (1) it should be a case-control, cross-sectional, or cohort study in humans; (2) it can be published in any language, but it must be a full-text paper in an international peer-reviewed journal before December 31, 2015; (3) there was no restriction on cancer type, but it must report adequate information on genotype frequencies to estimate ORs for the cancer type. Case reports, editorials, meta-analyses, and review articles were excluded.

A systematic literature search was conducted in Electronic databases, including PubMed, Web of Science, EMBASE, and Chinese National Knowledge Infrastructure (including China Doctoral/Master Dissertation Full-text Database, China Academic Journals Full-text Database, Century Journals Project, and China Proceedings of Conference Full-text Database), before December 31, 2015. We used the keywords: “(atm OR ataxia telangiectasia mutated) AND (polymorphism* OR variant* OR mutant* OR genotype*)”, in the searching process. This search yielded 3816 articles.

To achieve adequate statistical power for the meta-analysis on gene-environment interactions in carcinogenesis, eligible polymorphisms were those reported by more than three data sources of radiation exposure. For this purpose, we employed a two-stage screen strategy (Figure 1). First, we collected articles on the association between *ATM* genetic polymorphisms and cancer risk in individuals in the presence of radiation exposure. After screened by title, abstract, or full text if necessary, we identified 6 articles including 17 polymorphisms. References from the relevant articles or reviews were also searched for additional studies. This search yielded no extra articles. Finally, we found that rs1801516 was the only *ATM* polymorphism investigated by more than 3 articles. Second, we collected articles on the association between the rs1801516 polymorphism and cancer risk in individuals in the absence of radiation exposure. We included all surrogates of the rs1801516 polymorphism, including rs52821794, rs60879649, rs17503060 (http://www.ncbi.nlm.nih.gov/snp/), and rs4988023 [61]. Our search on Chinese National Knowledge Infrastructure database identified no article on the rs1801516 polymorphism and cancer risk (possibly due to a low MAF of < 0.05 in Asians [25, 62]. If different articles reported on the same sample, only the most complete information was included. If an article included multiple sources or study populations, data were extracted separately if possible. The article by Akulevich et al. studied radiation exposed populations as well as unexposed populations, the results for each group were considered as a separate study [12]. Finally, 29 studies without radiation exposure were identified to meet the inclusion criteria for subsequent quality assessment (Figure 1).

### Data collection

Two authors independently extracted data based on a standardized form. The following information was collected from each study: first author, year of publication, country of origin, ethnicity, family history of cases (familial cancer or sporadic cancer), MAF in controls, controls in HWE, cancer site, and number of genotyped cases and controls. Ethnicity was classified as African-American, Amerindian, Asian, Caucasian, or others based on the ethnicity of at least 80% of the study population [63]. When a study did not state the included ethnic groups, we considered the ethnicity of the source population based on the country where the study was performed [63]. When an article reported data for different ethnic groups, the results for each group were considered as a separate study. If it was impossible to separate participants according to ethnicity, the participants were considered as “others”. Study authors were contacted when there was insufficient information. Disagreement was resolved by discussion between authors.

### Quality assessment

Two authors independently evaluated the quality of each study, with discrepancies resolved during a consensus meeting. We performed two types of quality assessments. The first one was the assessment of methodological errors. Deviation from HWE in controls is an indication of a genotyping error or selection bias [64, 65], and was considered as a methodological error. Because including studies with methodological errors may lower the quality of evidence in a meta-analysis [66], these studies were excluded. However, it should be noted: (1) in case-only studies, HWE deviations may reflect an association with the disease, rather than poor genotyping [67]; (2) studies with insufficient information to determine whether the controls were in HWE were eligible for a meta-analysis, but the influence of these studies on the pooled result was examined in subgroup analyses. Second, the quality of each study was assessed according to the NOS specific to case-control study [45]. The NOS evaluates the quality of a study in three domains: selection, comparability, and exposure. For each study, a maximum score of 4 is assigned for selection, 2 for comparability, and 3 for exposure. A study is considered low (or high) quality if total NOS score is < 6 (or ≥ 6). Because the NOS score is a continuum, distinction between high and low quality is inevitably arbitrary. Due to the subjective nature, the NOS score was used as a stratification factor in the subgroup analysis to evaluate whether the results of the meta-analysis depended on the quality of the included studies [68].

### Procedures of meta-analyses

To clarify whether there was a joint effect between the rs1801516 polymorphism and radiation exposure in carcinogenesis, we performed three steps: 1). meta-analysis of the rs1801516 polymorphism and cancer risk in individuals in the presence of radiation exposure; 2). meta-analysis of the rs1801516 polymorphism and cancer risk in individuals in the absence of radiation exposure; 3). comparison of the differences in the effect estimates of the rs1801516 polymorphism on cancer risk between the two groups.

Subgroup meta-analyses were conducted based on pre-specified interests, including cancer site, ethnicity, familial cancer history, study quality, sample size, and HWE in controls. The criteria for a subgroup analysis required at least 3 studies. We aimed at determining whether the result of the overall meta-analysis was stable or dependent on some features of the included studies. Sensitivity analysis was conducted by excluding 1 study at a time and analyzing the remaining ones to explore whether the result was influenced by a particular study.

### Statistical analysis

ORs and 95% CIs were used to assess the strength of the association between cancer risk and the rs1801516 polymorphism. The ORs were calculated under four genetic models: (1) heterozygous model (AG versus GG), (2) homozygous model (AA versus GG), (3) dominant model (AA+AG versus GG), and (4) recessive model (AA versus AG+GG). The statistical significance of the ORs was evaluated by using the Z test. In case of zero cells, an appropriate continuity correction (addition of 0.5 in all the cells) was implemented [69]. Between-study heterogeneity was evaluated by using the Cochrane Q test and the I^2^ statistic. We used the random effects model (DerSimonian and Laird's method [70]) to calculate the ORs when the *P value* of the Cochrane Q test was < 0.10 or the I^2^ value was > 50%; otherwise, the fixed effects model was applied. The test of interaction proposed by Altman et al. [71] was used to compare differences in effect estimates between subgroups. When there were more than 10 studies in a meta-analysis, we estimated publication bias by visualizing funnel plots and by Egger's linear regression test [72] and Begg's rank correlation test [73]. To assess deviation from HWE, we performed the appropriate goodness-of-fit χ2 test. The above statistical analyses were performed by using Stata, version 12, software (StataCorp LP, College Station, Texas) with 2-sided *P value*s. Statistical significance was defined as *P* < 0.05.

## SUPPLEMENTARY MATERIALS FIGURE AND TABLE


