# Supplementary material for: Gene-environment interaction for polymorphisms in ataxia telangiectasia-mutated gene and radiation exposure in carcinogenesis: results from two literature-based meta-analyses of 27120 participants
Source: Oncotarget. 2016 Oct 18;7(47):76867–81. doi: 10.18632/oncotarget.12724 (PMC5363555; doi:10.18632/oncotarget.12724)
Supplement: Supplementary file 1 [file oncotarget-07-76867-s001.pdf]

# Gene-environment interaction for polymorphisms in ataxia telangiectasia-mutated gene and radiation exposure in carcinogenesis: results from two literature-based meta-analyses of 27120 participants

## Supplementary Materials

**Supplementary Table S1: Quality assessment of eligible studies using the newcastle–ottawa scale**

| First author, year [Ref.] | Selection | Comparability | Outcome | Total scores |
|---|---|---|---|---|
| Maillet P, 2000, [44] | 2 | 2 | 1 | 5 |
| Buchholz TA, 2004, [43] | 4 | 1 | 2 | 7 |
| Heikkinen K, 2005, [42] | 3 | 2 | 3 | 8 |
| Gonzalez-Hormazabal P, 2008, [41] | 2 | 2 | 1 | 5 |
| Angele S, 2003, [40] | 3 | 2 | 2 | 7 |
| Renwick A, 2006, [39] | 3 | 1 | 2 | 6 |
| Angele S, 2004, [38] | 4 | 1 | 3 | 8 |
| Yang H, 2007, [37] | 3 | 2 | 3 | 8 |
| Tommiska J, 2006, [36] | 3 | 1 | 2 | 6 |
| Wu X, 2006, [35] | 3 | 1 | 2 | 6 |
| Sommer SS, 2002, [34] | 2 | 2 | 2 | 6 |
| Xu L, 2012, [33] | 3 | 1 | 2 | 6 |
| Oliveira S, 2012, [17] | 2 | 2 | 1 | 5 |
| Margulis V, 2008, [32] | 3 | 2 | 2 | 7 |
| Al-Hadyan KS, 2012, [31] | 2 | 1 | 2 | 5 |
| Schrauder M, 2008, [30] | 4 | 1 | 2 | 7 |
| Dork T, 2001, [29] | 3 | 1 | 2 | 6 |
| Wojcicka A, 2014, [28] | 3 | 2 | 1 | 6 |
| Kristensen AT, 2004, [27] | 2 | 1 | 2 | 5 |
| Hirsch AE, 2008, [26] | 3 | 1 | 2 | 6 |
| Bretsky P, 2003, [25] | 4 | 2 | 2 | 8 |
| Pereda CM, 2015, [24] | 2 | 1 | 2 | 5 |
| Tecza K, 2015, [23] | 2 | 1 | 2 | 5 |
| Meier M, 2005, [22] | 2 | 1 | 2 | 5 |
| Maillard S, 2015, [16] | 3 | 2 | 2 | 7 |
| Akulevich NM, 2009, [12] | 4 | 1 | 2 | 7 |
| Damiola F, 2014, [15] | 4 | 1 | 2 | 7 |
| Broeks A, 2008, [13] | 3 | 2 | 2 | 7 |
| Concannon P, 2008, [14] | 4 | 2 | 2 | 8 |
| Offit K, 2002, [11] | 3 | 2 | 2 | 7 |

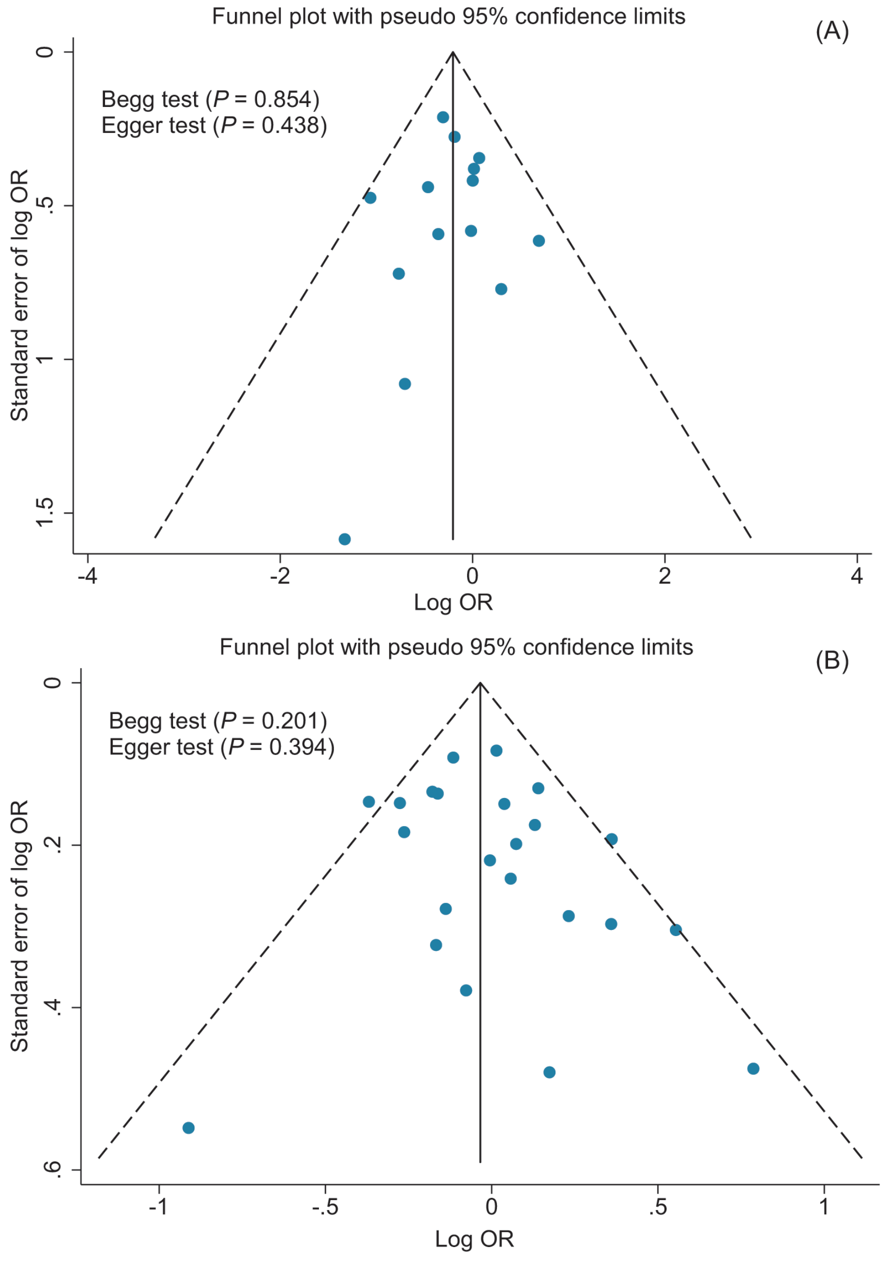


**Supplementary Figure S1: Funnel plot for publication bias in meta-analysis of the effect of the *ATM* rs1801516 polymorphism on cancer risk in individuals without radiation exposure.** (**A**) Homozygous model. (**B**) Dominant model. Abbreviations: CI, confidence interval; OR, odds ratio.
